# Association of Dietary Retinol Intake and Serum Neurofilament Light Chain Levels: Results from NHANES 2013–2014

**DOI:** 10.3390/nu16111763

**Published:** 2024-06-04

**Authors:** Na Liang, Hao Li, Keyi Zhang, Yan Wang, Lu Xiang, Lin Xiao, Gang Luo

**Affiliations:** Xiangya School of Public Health, Central South University, Changsha 410078, China; 236912047@csu.edu.cn (N.L.); 236912060@csu.edu.cn (H.L.); zhangky@csu.edu.cn (K.Z.); 226911039@csu.edu.cn (Y.W.); xianglu66@csu.edu.cn (L.X.); xiaolinxl@csu.edu.cn (L.X.)

**Keywords:** dietary retinol intake, serum neurofilament light chain, NHANES, sNfL

## Abstract

Background: There is increasing evidence suggesting that serum neurofilament light chain (sNfL) levels can be used as biomarkers for axonal injury. Retinol is recognized for its significant involvement in nervous system function, but the precise connection between dietary retinol and sNfL levels remains uncertain. Objective: Our objective was to investigate the relationship between dietary retinol intake and sNfL, and to find an optimal retinol intake level for neurological health. Methods: In the National Health and Nutrition Examination Survey (NHANES), conducted from 2013 to 2014, a cohort of 1684 participants who met the criteria were selected for the study. sNfL levels were measured from stored serum samples using a novel high-throughput immunoassay platform from Siemens Healthineers. Assessment of dietary retinol intake was performed by a uniformly trained interviewer through a 24 h dietary recall method. A generalized linear model was evaluated to assess the correlation between dietary retinol intake and sNfL concentrations. Furthermore, the nonlinear association between the two is further explored using restricted cubic spline (RCS) analysis. Results: Upon adjusting for potential confounders, a 10% increase in dietary retinol intake was associated with a 3.47% increase in sNfL levels (95% CI: 0.54%, 6.49%) across all participants. This relationship was more pronounced in specific subgroups, including those under 60 years of age, non-obese, impaired estimated glomerular filtration rate (eGFR), and non-diabetic. In subgroup analysis, among those younger than 60 years of age (percent change: 3.80%; 95% CI: 0.43%, 7.28%), changes were found in non-obese participants (percent change: 6.28%; 95% CI: 2.66%, 10.02%), those with impaired eGFR (percent change: 6.90%; 95% CI: 1.44%, 12.65%), and non-diabetic patients (percentage change: 4.17%; 95% CI: 1.08%, 7.36%). RCS analysis showed a linear relationship between dietary retinol intake and sNfL levels. Furthermore, the positive correlation between the two was more significant after the inflection point, according to piecewise linear analysis. Conclusion: This current investigation uncovered a J-shaped relationship between dietary retinol and sNfL levels, suggesting that axonal damage can occur when dietary retinol intake increases more than a specific threshold. These findings need to be further confirmed in future prospective studies to determine the precise intake level that may trigger axonal injury.

## 1. Introduction

Neurofilament fibrin is a significant component of the neuronal cytoskeleton and is only expressed in mature nerve cells [1]. Neurofilament light chain (NfL) protein is one of the major subunits of neurofilaments, and under normal circumstances, NfL is undetectable in the blood [2]. However, during axon damage, NfL proteins are released in substantial quantities into the cerebrospinal fluid and eventually into the blood [3]. Elevated serum neurofilament light chain (sNfL) levels have been observed in various neurodegenerative diseases, and measurements of sNfL levels can serve as a reflection of dynamic changes in the degree of axonal damage and disease progression [4,5,6].

Vitamin A (VA), an essential fat-soluble vitamin for human growth and development, encompasses a group of compounds with biological activity, including retinol and carotenoids [7]. VA deficiency is recognized as a significant global micronutrient deficiency disease [8]. As the main active substance of VA, retinol cannot be synthesized endogenously by the body and is mainly consumed through animal foods [9]. According to the Dietary Guidelines for Americans, individuals over the age of 14 are advised to consume 700 retinal activity equivalents (RAEs) for women and 900 RAEs for men on a daily basis. After entering the liver through the digestive tract, retinol binds to retinol-binding protein (RBP) and enters the plasma with transthyroxine (TTR) to form the retinol–RBP–TTR complex, facilitating retinol transport to target organs. In addition to being an important retinol carrier protein, TTR also plays a protective role in the nervous system. It has been demonstrated that TTR is involved in behavior, cognition, amidation, neuropeptide processing, and nerve regeneration [10]. Furthermore, TTR has been found to have neuroprotective effects in Alzheimer’s disease (AD) and cerebral ischemia [11]. The breakdown of TTR is considered a necessary step in central nervous system amyloidosis [12]. Recent investigations suggest that retinol can stabilize the structure of TTR and significantly reduce its decomposition rate through interaction with RBP [13]. The hippocampus is among the few areas of the brain that generate new neurons after birth, and a lack of VA reduces the ability of new neurons to develop and survive [14,15]. An intriguing study has demonstrated that VA deficiency can lead to hippocampal memory impairment and learning and memory deficits in rats [16]. Studies have shown that VA mainly plays a role in hippocampal memory through retinoic acid, a metabolite of retinol [17]. Consequently, the amount of retinol in the body plays an important role in maintaining the normal function of the nervous system of the brain.

There is compelling evidence indicating that sNfL levels are highly stable and represent one of the most promising biomarkers for clinical brain injury or neurodegenerative conditions [18,19]. Notably, baseline sNfL concentrations were found to predict multiple sclerosis (MS) progression in a European longitudinal MS cohort [6]. The study found that sNfL was elevated in plasma in patients with AD and mild cognitive impairment. Based on the significant impact of retinol on the nervous system, it is imperative to know whether dietary supplementation of retinol can mitigate damage to the nervous system. However, the relationship between dietary retinol intake and sNfL levels remains unclear. Therefore, we conducted an analysis utilizing data from the National Health and Nutrition Examination Survey (NHANES) between 2013 and 2014 to explore the association between dietary retinol intake and sNfL levels.

## 2. Materials and Methods

### 2.1. Study Population Samples

The NHANES, established in 1960, is a cross-sectional survey conducted by the Centers for Disease Control to assess the health and nutrition status of the entire population of the United States. Since 1999, this program has conducted biennial surveys with a nationally representative sample size of approximately 5000 individuals surveyed annually. Informed consent was obtained from all study participants, and the ethics protocol was approved by the National Center for Health Statistics Review Committee. For more comprehensive information about NHANES, please visit their official website (https://www.cdc.gov/nchs/nhanes/index.htm, accessed on 23 April 2024).

**Figure 1:**
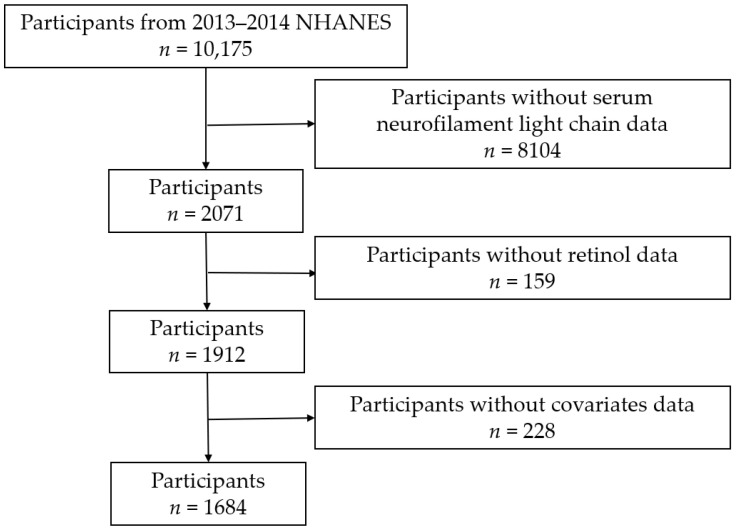
Flowchart of the study population.

This investigation utilized NHANES 2013–2014 cycle data, as depicted in Figure 1. A total of 10,175 participants were included in this cycle. After excluding samples with missing sNfL data (*n* = 8104) and samples lacking dietary retinol data (*n* = 159) and further eliminated participants with incomplete covariates (*n* = 228). Covariates considered for exclusion comprised age, sex, body mass index (BMI), poverty income ratio (PIR), education, ethnicity, smoking habits, alcohol consumption, diabetes status, estimated glomerular filtration rate (eGFR), metabolic equivalent (MET), and extreme dietary energy intake (<800 or >4200 kcal/day for males and <600 or >3500 kcal/day for females). Ultimately, a total of 1684 participants were enrolled in this study.

### 2.2. Dietary Retinol Intake Assessment

The dietary intake information for NHANES was obtained through two 24 h dietary recall interviews with all participants, conducted in collaboration with the U.S. Department of Agriculture (USDA) and the U.S. Department of Health and Human Services. Trained interviewers utilized the Automated Multiple Pass Method tool provided by USDA to ensure data accuracy and consistency across different interview stages and quality control measures. The initial recall took place face-to-face at a mobile examination center, where participants were equipped with a measurement tool kit containing various utensils and a food model booklet to facilitate precise reporting of food intake. Subsequently, a second recall was conducted via telephone 3–10 days later. This approach involving dual dietary recalls enabled estimation of the types and quantities of foods and beverages consumed by participants within 24 h prior to each interview, allowing for the estimation of retinol consumption from these sources (μg/day).

### 2.3. Measurement of sNfL Levels

In this study, half of the participants aged 20–75 years provided consent for blood samples for analysis. The blood samples were analyzed using acoustic emission technology on the Attelica immunoassay system, which employs acridol chemiluminescence and paramagnetic particles to improve sensitivity and speed during the sNfL immunoassay process. Initially, the sample is incubated with acridinium-ester (AE)-labeled antibodies that bind to the NfL antigen. Subsequently, paramagnetic particles (PMPs) coated with capture antibodies are introduced to form complexes of antigens bound to AE-labeled antibodies and PMP. Unbound AE-labeled antibodies are then isolated and removed, followed by the addition of acid and base to initiate chemiluminescence, with subsequent measurements of light emission. Rigorous adherence to quality assurance procedures was upheld throughout the analysis and measurement processes. In addition to study samples, low-, medium-, and high-concentration quality control samples were run every 8 h shift along with additional replicate samples in order to ensure the accuracy and reliability of the derived data. The detection range of sNfL is 3.9–500 pg/mL.

### 2.4. Covariates

Several covariates were identified through the literature, including age, sex, race, education, BMI, PIR, smoking, alcohol consumption, diabetes, eGFR, MET, and energy intake [20,21,22]. Race is classified as non-Hispanic white, non-Hispanic black, other Hispanic, Mexican American, or other. The level of education is defined as lower than high school level, high school education, university, and above. BMI is calculated by dividing weight by the square of height. Those who have had at least 100 cigarettes in their lifetime are classified as smoking and the rest as not smoking. Alcohol consumption is defined as having at least 12 alcoholic drinks per year, with the rest classified as non-drinkers. Diabetes is defined as having been diagnosed with diabetes by a doctor, currently taking insulin or diabetes medications, with an HbA1c level of ≥6.5% or a fasting blood glucose level of ≥126 mg/dL, and an oral glucose tolerance test 2 h > 11.1 mmol/L. The formula for calculating the glomerular filtration rate is eGFR = 141 × min (SCr/κ, 1)^α^ × max (SCr/κ, 1)^−1.209^ × 0.993^age^ × 1.018 [if female] × 1.159 [if black] [23]. Physical activity was calculated as METS for each participant using the recommended MET score in the NHANES (8.0 for vigorous work or recreational activity; moderate work or recreation activity is a score of 4.0; sitting is 1.5 points).

### 2.5. Statistical Analysis

Statistical analysis was performed according to the recommended NHANES analysis guidelines, utilizing reweighted estimates. sNfL levels were transformed by the natural logarithm to satisfy the normal distribution. Continuous variables with normal distribution are expressed as mean ± standard deviation (SD), continuous variables with skewed distributions are described in terms of medians (interquartile intervals), and categorical variables are expressed as frequencies (percentages). ANOVA was used to compare continuous variables, and the chi-square test was used for categorical variables. Dietary retinol intake, treated as a constant variable, was divided into four quartiles and could also be analyzed as a categorical variable.

A generalized linear model was used to assess the association between dietary retinol intake and sNfL levels, adjusting for potential covariates based on the literature review. In order to effectively control confounding factors, three regression models were constructed in this study. Model 1 was the original model without adjustment; Model 2 adjusted for age and sex; Model 3 further adjusted for race, education, PIR, BMI, smoking, alcohol consumption, diabetes, eGFR, dietary energy intake, and MET. To enhance the interpretation of the regression model, we estimated the percentage change in sNfL with increasing dietary retinol intake based on the following formula: (e^(IQR×β)^ − 1) × 100%, and 95% CI: (e [IQR × (β ± 1.96 × SE)] − 1) × 100%. Here, β is the regression coefficient, SE is the standard error, and IQR is the interquartile range.

The nonlinear correlation between dietary retinol intake and sNfL concentration was further investigated by restricted cubic spline (RCS) analysis with four knots placed at the 5th, 35th, 65th, and 95th percentiles. Based on previous studies showing associations between age [24], gender [25], BMI [26], eGFR [27], and diabetes [28] and sNfL levels, we conducted subgroup analyses to explore the age differences (<60 years; ≥60 years old), gender (male; female), BMI (non-obese: <30 kg/m^2^; obesity: ≥30 kg/m^2^), eGFR (normal: ≥90 mL/min/1.73 m^2^; impairment: <90 mL/min/1.73 m^2^) and diabetes (absent; present). In each subgroup, we examined the relationship between dietary retinol intake and sNfL concentration, treating the median dietary retinol intake within each quartile as a continuous variable. The linear trend test was used to evaluate correlations with sNfL levels, and the Wald test was used to introduce the interaction effect. R software (version 4.3.1) was used for both exclusion and statistical analysis of NHANES data, and the difference was considered statistically significant when bilateral *p* < 0.05.

## 3. Results

### 3.1. Baseline Characteristics for All Participants

Table 1 displays the basic characteristics of the participants. A total of 1684 participants participated in the study, with a mean age of 46.59 ± 15.50 years old, including 812 males (48.2%) and 872 females (51.8%). The average dietary intake of retinol was 400.24 ± 385.23 μg/d. In terms of racial distribution, nearly half of the participants were non-Hispanic white. The majority of participants were well educated and had a college degree or higher. Participants did not differ in age, BMI, educational attainment, PIR, smoking, alcohol consumption, eGFR, diabetes, or MET.

### 3.2. Association between Retinol Intake and sNfL Levels

The relationship between dietary retinol intake and sNfL levels is demonstrated in Table 2. There was a significant association between sustained dietary retinol intake and sNfL levels, even after adjusting for potential confounders. In the unadjusted crude model, for every 10% increase in dietary retinol intake among all study participants, sNfL levels increased by 3.62% (95% CI: 0.52%, 6.81%). After adjusting for age and sex, sNfL levels increased by 3.38% for every 10% increase in dietary retinol intake (95% CI: 0.67%, 6.17%). In Model 3, after further adjustment for covariates such as race, BMI, PIR, education, smoking, alcohol consumption, diabetes, eGFR, MET, and dietary energy intake, there was a significant positive association (percentage change: 3.47%; 95% CI: 0.54%, 6.49%).

**Figure 2:**
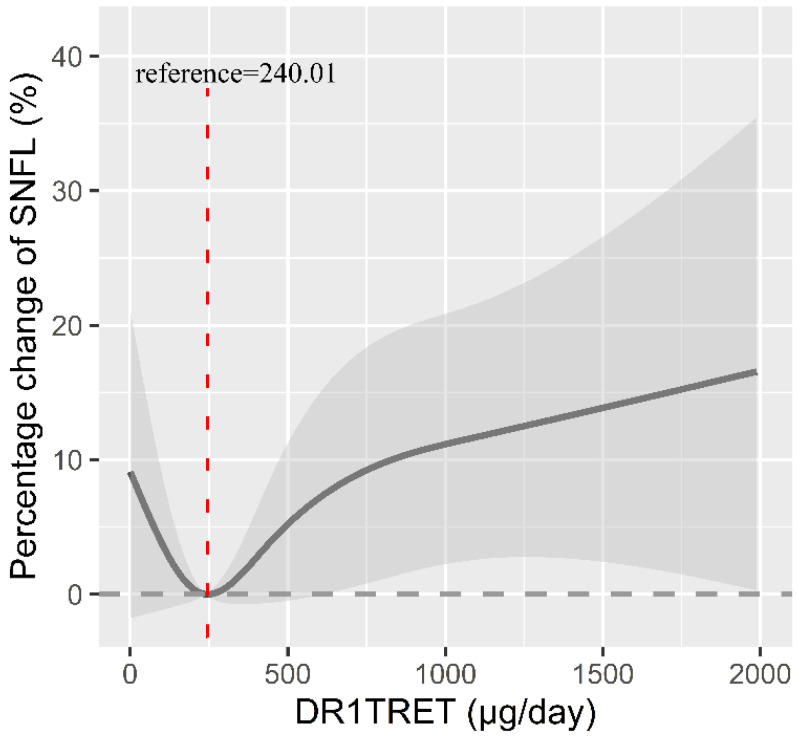
Dose–response relationship between retinol intake and serum neurofilament light chain levels in 1684 participants. Restricted cubic splines were created at the 5th, 35th, 65th, and 95th percentiles of percent retinol intake at 4 nodes. The solid line is a dot estimate of the association between retinol intake and serum neurofilament light chain, the grey shaded part is a 95% CI estimate, and the red dotted line is the inflection point reference value. The model was adjusted for age, sex, BMI, PIR, education, ethnicity, smoking, alcohol consumption, diabetes, eGFR, dietary energy intake, and exercise. *p* for nonlinearity is 0.137.

Furthermore, we validated the dose–response relationship between dietary retinol intake and sNfL levels using RCS. The dose relationship between retinol intake and sNfL was J-shaped (Figure 2). However, overall, there was no deviation from linearity among all participants (nonlinearity *p* = 0.137). Additionally, a threshold effect analysis showed that before the inflection point, the correlation between dietary retinol intake and sNfL levels was low and not statistically significant. However, after the inflection point, a significant positive correlation was observed between the dietary retinol intake (per 10% increment) and sNfL levels (percentage change = 4.39%, 95% CI: 0.89%, 8.01%, *p* < 0.05) (Table 3). In addition, RCS was used to estimate the dose–response relationship between dietary retinol intake and sNfL levels in each subgroup (Supplementary Figures S1–S10). The results showed a significant linear relationship between dietary retinol intake and sNfL levels in patients aged < 60, non-diabetic, non-obese participants, and renal-impaired participants.

### 3.3. Subgroup Analysis

The current study conducted subgroup analyses to explore whether the relationship between dietary retinol intake and sNfL levels was influenced by important factors such as age, sex, BMI, eGFR, and diabetes. The results are presented in Table 4. After adjusting for potential confounders, a significant association was observed between dietary retinol intake and sNfL levels in participants of age < 60, non-obese individuals, participants with impaired renal function, and participants without diabetes. Specifically, for every 10% increase in dietary retinol intake, sNfL levels increased by 3.80% in those aged < 60 (95% CI: 0.43%, 7.28%), 6.28% in non-obese subjects (95% CI: 2.66%, 10.02%), 6.90% in renal-impaired subjects (95% CI: 1.44%, 12.65%), and 4.17% in non-diabetic subjects (95% CI: 1.08%, 7.36%).

## 4. Discussion

In this study, our objective was to assess the relationship between dietary retinol and sNfL concentrations in a large, representative cohort of U.S. adults comprising 1684 participants. The findings revealed a significant linear positive association between dietary retinol intake and sNfL concentrations across all participants. This positive correlation was particularly pronounced among participants under the age of 60, non-obese individuals with impaired eGFR, and those without diabetes.

sNfL serves as a well-established biomarker for various neurodegenerative disorders, including MS, and has been observed to have a close relationship with disease progression and prognosis [29,30,31,32,33,34,35]. While diet plays a crucial environmental factor in influencing the health of the nervous system, research in this domain remains limited. Markus Bock and colleagues have demonstrated the impact of caloric restriction and adaptive ketogenic diets (KDs), which can impact neuroinflammation in MS and other neurological conditions [36]. Furthermore, Unsong Oh et al. have noted that KDs can contribute to maintaining lower and more stable sNfL levels in patients with relapsing MS, suggesting a promising therapeutic strategy for managing this condition [37]. Recent literature emphasizes the neuroprotective role of vitamin D (VD) in neuropsychiatric disorders. Evidence from multiple studies suggests that VD supplementation may mitigate the onset and symptomatology of these conditions [38,39,40,41]. However, Trygve Holøy’s clinical trial found that high-dose VD supplements did not reduce sNfL levels in MS patients [42]. This result was also confirmed in Katariina Hanninen’s controlled clinical trial [43]. Billy R Hammond discovered in 2015 that carrots may play an essential role in the nervous system and perform multiple functions in the central nervous system [44]. Marcos Roberto de Oliveira points out that excessive VA supplementation is potentially neurotoxic, especially in individuals with neurodegenerative diseases, emphasizing the importance of appropriate dosing to avoid adverse health consequences [45]. VA is an essential vitamin for maintaining human growth and development, and its lack and excess will seriously harm our health; accordingly, it is important to explore a suitable dose. Our study contributes to refining the understanding of the relationship between VA and the nervous system by analyzing the association between dietary retinol intake and sNfL levels.

Our results indicate a significant positive association between retinol intake and sNfL levels. The J-type dose–response curve revealed that sNfL levels decreased with increasing retinol intake in subjects with dietary retinol intake below 240.01 μg, although this association was not statistically significant. However, sNfL levels increased significantly with increasing retinol intake in subjects with retinol intake exceeding 240.01 μg. The potential mechanism underlying the relationship between dietary retinol intake and serum sNfL levels may involve RBP and TTR. TTR plays a neuroprotective role at a certain concentration in the body [10,11], but its breakdown is essential in central nervous system amyloidosis [12]. Recent research indicates that retinol may stabilize TTR’s structure and decrease its decomposition rate by binding to RBP [13]. However, excessive levels of retinol have been shown to negatively regulate the expression of RBP receptors, leading to a decrease in the binding and transport of retinol with RBP and potentially accelerating the breakdown of TTR [10]. Additionally, when retinol is excessive, it could induce oxidative damage to the brain.

The positive association between retinol intake and sNfL levels was found to be more pronounced in people under 60 years of age, non-obese individuals, people with impaired eGFR, and those without diabetes. The reason may be the abundance of NfL in younger age groups, while sNfL levels after age 60 are much less influenced by diet than age [46]. Obesity can cause metabolic dysfunction, dyslipidemia, and inflammation, which lead to various diseases and damage to the nervous system [47]. Mazon Janaina Niero has shown that obesity does lead to neurodegeneration [48]. Long-term hyperglycemia in diabetic patients promotes the release of inflammatory factors and damages the integrity of the blood–brain barrier, and NFL is released into the blood through the damaged blood–brain barrier, resulting in neuropathy [49]. Therefore, the association between retinol intake and sNfL levels in older adults, obese, and diabetic individuals may be masked by the effects of age and obesity on sNfL.

A study found that individuals or groups with poor kidney function tend to have higher plasma NfL than those with good kidney function [50]. This is consistent with our observation that individuals with impaired renal function are more susceptible to the effects of retinol on sNfL. It has previously been reported that estrogen has a neuroprotective effect and that oral estrogen can reduce sNfL levels in women with MS, but we did not observe a gender difference between retinol intake and sNfL levels [51]. Hence, it is imperative that future longitudinal studies be conducted to ascertain the presence of a sex disparity in the correlation between dietary retinol intake and sNfL levels. Furthermore, additional research on the involvement of sNfL in neurosystem-related disorders and the influence of dietary retinol on sNfL expression will aid in the formulation of health initiatives focused on averting neurological harm from a nutritional standpoint.

Although we observed a strong positive association between retinol intake and sNfL levels in the overall population, it was also validated across different subgroups. However, given that we only have a sample of sNfL data from 2013 to 2014 and that NHANES is a cross-sectional design, we cannot speculate on causal or temporal associations, highlighting limitations in our study. Finally, while we account for as many covariates as possible, we do not exclude the possibility that some unmeasured or unrecorded confounding factors may still influence the results.

## 5. Conclusions

The present investigation revealed a J-shaped relationship between dietary retinol intake and sNfL levels, and the inflection point was about 240.01 μg. When the intake exceeded 240.01 μg, there was a significant positive correlation between dietary retinol intake and sNfL levels. It was more evident in people under 60 years of age, non-obese people, people with impaired GFR, and people without diabetes. These findings show that the intake of appropriate dietary retinol is beneficial to the health of the nervous system function while simultaneously highlighting the potential neurotoxicity of excessive retinol intake.

## Figures and Tables

**Table 1:** Basic characteristics of NHANES participants (N = 1684).

Characteristic	All	Quartile of Dietary Retinol Intake (μg/day)				
		Quartile 1	Quartile 2	Quartile 3	Quartile 4	*p*-Value
N	1684	420	421	419	424	
Age, years (mean (SD))	46.59 ± 15.5	46.19 (15.6)	47.05 (15.5)	47.18 (15.4)	45.93 (15.4)	0.569
Sex, n (%)						<0.001
male	812 (48.2)	189 (45.0)	168 (39.9)	189 (45.1)	266 (62.7)	
female	872 (51.8)	231 (55.0)	253 (60.1)	230 (54.9)	158 (37.3)	
BMI, kg/m^2^ (%)						0.345
<30 kg/m^2^	1040 (61.8)	275 (65.5)	256 (60.8)	252 (60.1)	257 (60.6)	
≥30 kg/m^2^	644 (38.2)	145 (34.5)	165 (39.2)	167 (39.9)	167 (39.4)	
Race, n (%)						<0.001
Mexican American or other	447 (26.6)	135 (32.1)	125 (29.7)	110 (26.3)	77 (18.2)	
Non-Hispanic black	302 (17.9)	104 (24.8)	61 (14.5)	69 (16.5)	68 (16.0)	
Non-Hispanic white	783 (46.5)	137 (32.6)	203 (48.2)	200 (47.7)	243 (57.3)	
Other Hispanic	152 (9.0)	44 (10.5)	32 (7.6)	40 (9.5)	36 (8.5)	
Education, n (%)						0.545
<High school	93 (5.5)	29 (6.9)	19 (4.5)	24 (5.7)	21 (5.0)	
High school	233 (13.8)	58 (13.8)	59 (14.0)	65 (15.5)	51 (12.0)	
College or above	1358 (80.6)	333 (79.3)	343 (81.5)	330 (78.8)	352 (83.0)	
PIR (mean (SD))	2.54 ± 1.7	2.38 (1.6)	2.62 (1.7)	2.53 (1.7)	2.63 (1.7)	0.119
Smoke (%)						0.837
No	949 (56.4)	237 (56.4)	238 (56.5)	229 (54.7)	245 (57.8)	
Yes	735 (43.6)	183 (43.6)	183 (43.5)	190 (45.3)	179 (42.2)	
Alcohol (%)						0.879
No	422 (25.1)	110 (26.2)	107 (25.4)	104 (24.8)	101 (23.8)	
Yes	1262 (74.9)	310 (73.8)	314 (74.6)	315 (75.2)	323 (76.2)	
Diabetes (%)						0.997
No	1384 (82.2)	344 (81.9)	347 (82.4)	345 (82.3)	348 (82.1)	
Yes	300 (17.8)	76 (18.1)	74 (17.6)	74 (17.7)	76 (17.9)	
eGFR, mL/min/1.73 m^2^ (mean (SD))	96.05 ± 21.4	96.79 (21.13)	96.06 (21.39)	95.95 (22.19)	95.41 (20.75)	0.825
MET (mean (SD))	638.6 ± 2638.8	572.89 (815.9)	543.99 (839.0)	556.89 (795.8)	878.24 (5063.8)	0.195
DR1TKCAL (mean (SD))	2172.03 ± 1037.0	1608.65 (789.6)	1975.81 (800.0)	2252.26 (840.0)	2845.65 (1230.2)	<0.001
DR1TRET (mean (SD))	309 (374)	74.39 (43.96)	229.26 (45.09)	405.03 (63.69)	888.06 (457.65)	<0.001
SNFL (median [IQR])	16.6 ± 20.6	11.55 [8.0, 18.7]	12.30 [8.1, 19.0]	12.40 [8.1, 17.8]	12.50 [8.7, 19.7]	0.388

Note: Values are presented as the mean ± SD, median (interquartile range), or n (%). SD: standard deviation; BMI, body mass index; PIR, family poverty income ratio; eGFR: estimated glomerular filtration rate; MET, metabolic equivalent.

**Table 2:** The association between the retinol intake and serum neurofilament light chain levels among all participants.

Models	Percentage Change (%) and 95% CI	*p*-Value
Model 1	3.62 (0.52, 6.81)	0.022
Model 2	3.38 (0.67, 6.17)	0.014
Model 3	3.47 (0.54, 6.49)	0.020

Note: CI: confidence interval. Model 1 was a crude model; Model 2 was adjusted for age and sex; Model 3 was adjusted for age, sex, BMI, PIR, education, ethnicity, smoking, alcohol consumption, diabetes, eGFR, dietary energy intake, and exercise.

**Table 3:** Threshold analyses of dietary retinol intake (per 10% increment) on the level of serum neurofilament light chain using two-piecewise regression models.

Retinol Intake (μg/day)	Crude Model		Adjusted Model	
	Percentage Change (%) and 95% CI	*p*-Value	Percentage Change (%) and 95% CI	*p*-Value
Fitting by two piecewise linear models				
≤240.01	−0.48 (−25.78, 33.45)	0.974	−14.21 (−34.23, 11.89)	0.258
>240.01	4.20 (0.35, 8.20)	0.032	4.39 (0.89, 8.01)	0.014

Note: CI: confidence interval. The adjusted model was adjusted for age, sex, BMI, PIR, education, ethnicity, smoking, alcohol consumption, diabetes, eGFR, dietary energy intake, and exercise.

**Table 4:** The association between the retinol intake and serum neurofilament light chain levels among subgroup participants.

Participants	N	Models	Percentage Change (%) and 95% CI	*p*-Value	*p* for Interaction
Age subgroup					0.535
<60	1246	Model 1	4.48 (1.19, 7.87)	0.007	
		Model 2	3.49 (0.20, 6.90)	0.038	
		Model 3	3.80 (0.43, 7.28)	0.027	
≥60	438	Model 1	3.72 (−2.28,10.08)	0.230	
		Model 2	4.60 (−1.72, 11.33)	0.158	
		Model 3	3.39 (−3.49, 10.78)	0.343	
Sex subgroup					
Male	812	Model 1	2.39 (−1.28, 6.21)	0.205	0.428
		Model 2	2.83 (−0.46, 6.22)	0.093	
		Model 3	3.04 (−0.56, 6.77)	0.100	
Female	872	Model 1	4.53 (−1.39, 10.80)	0.137	
		Model 2	4.72 (−0.21, 9.90)	0.061	
		Model 3	4.52 (−0.76, 10.07)	0.095	
BMI subgroup					0.115
Non-obese (<30 kg/m^2^)	1040	Model 1	5.68 (1.95, 9.54)	0.003	
		Model 2	5.30 (2.05, 8.65)	0.001	
		Model 3	6.28 (2.66, 10.02)	<0.001	
Obese (≥30 kg/m^2^)	644	Model 1	0.64 (−4.64, 6.20)	0.818	
		Model 2	0.67 (−3.95, 5.50)	0.781	
		Model 3	0.22 (−4.67, 5.37)	0.930	
eGFR subgroup					0.140
Healthy (≥90 mL/min/1.73 m^2^)	1076	Model 1	1.23 (−2.30, 4.88)	0.501	
		Model 2	0.10 (−3.11, 3.40)	0.954	
		Model 3	0.96 (−2.53, 4.59)	0.594	
Damaged (<90 mL/min/1.73 m^2^)	608	Model 1	6.16 (1.21, 11.35)	0.015	
		Model 2	8.56 (3.81, 13.52)	<0.001	
		Model 3	6.90 (1.44, 12.65)	0.013	
Diabetes subgroup					0.351
No	1384	Model 1	4.75 (1.49, 8.11)	0.004	
		Model 2	3.75 (0.91, 6.67)	0.009	
		Model 3	4.17 (1.08, 7.36)	0.008	
Yes	300	Model 1	−1.96 (−10.28, 7.12)	0.662	
		Model 2	0.65 (−7.64, 9.67)	0.883	
		Model 3	−4.59 (−13.33, 5.05)	0.340	

Note: CI: confidence interval. Model 1 was a crude model, including retinol intake; Model 2 was adjusted for age and sex; Model 3 was adjusted for age, sex, BMI, PIR, education, ethnicity, smoking, alcohol consumption, diabetes, eGFR, dietary energy intake, and exercise. The *p* for interaction is for the interaction of retinol intake with age, sex, BMI, eGFR, or diabetes.

## Data Availability

The datasets produced and examined during the current study can be openly accessed via the NHANES website (https://wwwn.cdc.gov/nchs/nhanes/Default.aspx, accessed on 23 April 2024).

## Supplementary Materials

The following supporting information can be downloaded at: https://www.mdpi.com/article/10.3390/nu16111763/s1, Figure S1: The relationship between dietary retinol and sNfL levels in participants aged < 60 years; Figure S2: The relationship between dietary retinol and sNfL levels in participants aged > 60 years; Figure S3: Relationship between dietary retinol and sNfL levels in male participants; Figure S4: Relationship between dietary retinol and sNfL levels in female participants; Figure S5: Association between dietary retinol and sNfL levels in participants with BMI < 30 kg/m^2^; Figure S6: Relationship between dietary retinol and sNfL levels in participants with BMI > 30 kg/m^2^; Figure S7: Association between dietary retinol and sNfL levels in participants without diabetes; Figure S8: The relationship between dietary retinol and sNfL levels in diabetic patients; Figure S9: Association between dietary retinol and sNfL levels in participants with healthy renal function; Figure S10: Association between dietary retinol and sNfL levels in participants with impaired renal function.
