# Supplementary material for: Association of Dietary Retinol Intake and Serum Neurofilament Light Chain Levels: Results from NHANES 2013–2014
Source: Nutrients. 2024 Jun 4;16(11):1763. doi: 10.3390/nu16111763 (PMC11175068; doi:10.3390/nu16111763)
Supplement: Supplementary file 1 [file nutrients-16-01763-s001.zip › nutrients-3003918-supplementary.pdf]

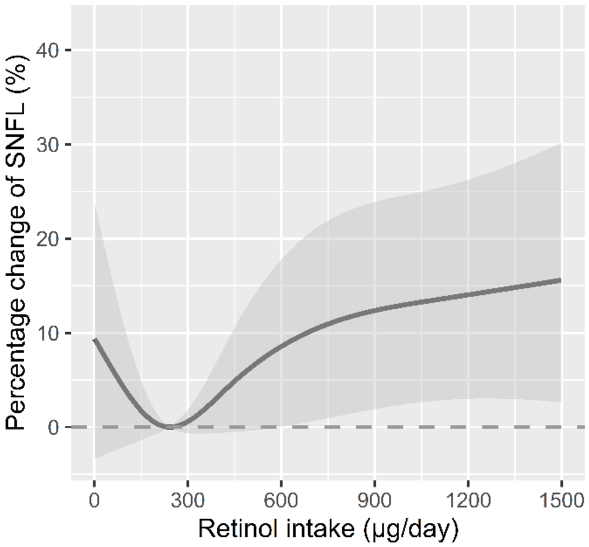

Figure S1. The relationship between dietary retinol and sNFL levels in participants aged <60 years. Restriction cubic spline curves were used at the 5th, 35th, 65th and 95th percentiles of dietary retinol intake. The shaded part represents the 95% CI interval. Models were adjusted for gender, BMI, PIR, education, ethnicity, smoking, alcohol consumption, diabetes, hypertension, eGFR, MET, and dietary energy intake.

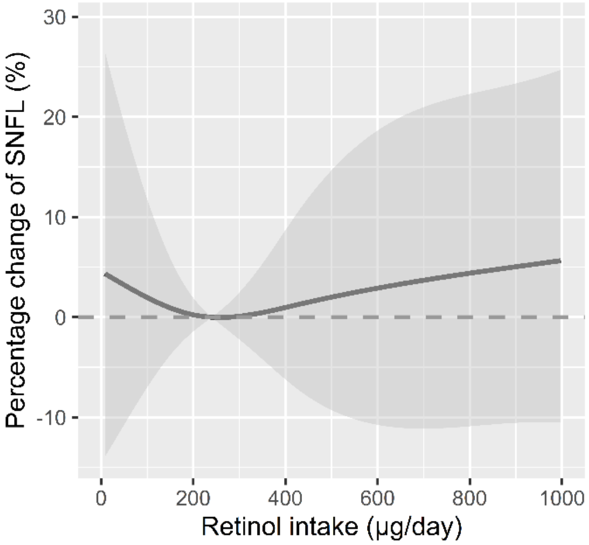

Figure S2. The relationship between dietary retinol and sNFL levels in participants aged >60 years. Restriction cubic spline curves were used at the 5th, 35th, 65th and 95th percentiles of dietary retinol intake. The shaded part represents the 95% CI interval. Models were adjusted for gender, BMI, PIR, education, ethnicity, smoking, alcohol consumption, diabetes, hypertension, eGFR, MET, and dietary energy intake.

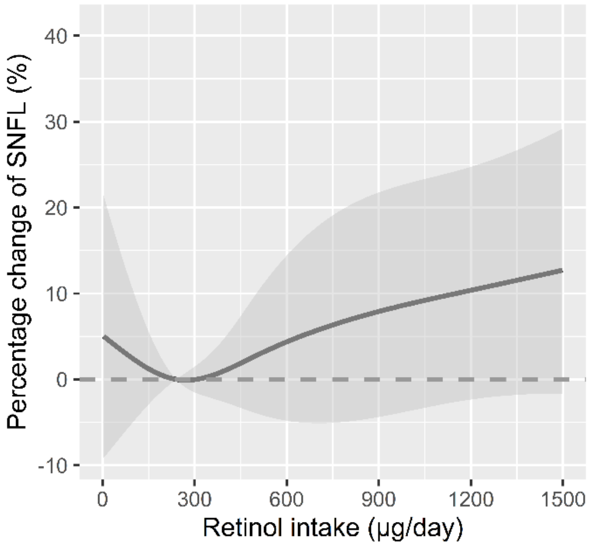

Figure S3. Relationship between dietary retinol and sNFL levels in male participants. Restriction cubic spline curves were used at the 5th, 35th, 65th and 95th percentiles of dietary retinol intake. The shaded part represents the 95% CI interval. Models were adjusted for age, BMI, PIR, education, ethnicity, smoking, alcohol consumption, diabetes, hypertension, eGFR, MET, and dietary energy intake.

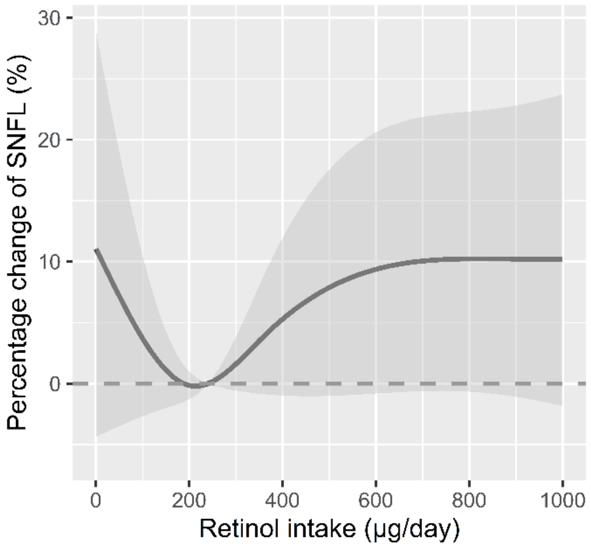

Figure S4. Relationship between dietary retinol and sNFL levels in female participants. Restriction cubic spline curves were used at the 5th, 35th, 65th and 95th percentiles of dietary retinol intake. The shaded part represents the 95% CI interval. Models were adjusted for age, BMI, PIR, education, ethnicity, smoking, alcohol consumption, diabetes, hypertension, eGFR, MET, and dietary energy intake.

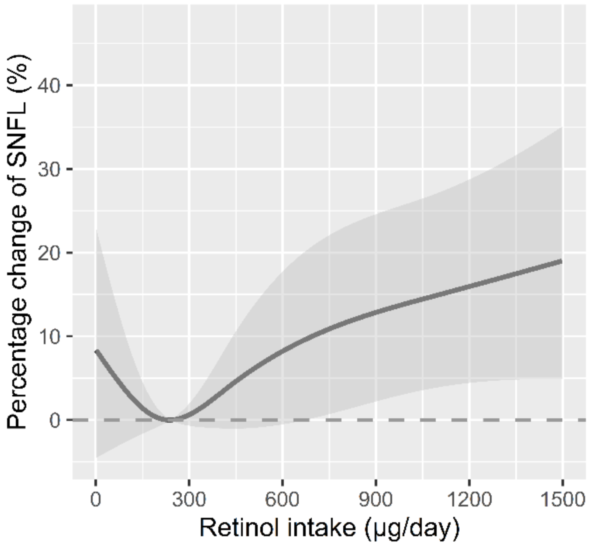

Figure S5. Association between dietary retinol and sNFL levels in participants with BMI<30 kg/m^2. Restriction cubic spline curves were used at the 5th, 35th, 65th and 95th percentiles of dietary retinol intake. The shaded part represents the 95% CI interval. Models were adjusted for age, gender, PIR, education, ethnicity, smoking, alcohol consumption, diabetes, hypertension, eGFR, MET, and dietary energy intake.

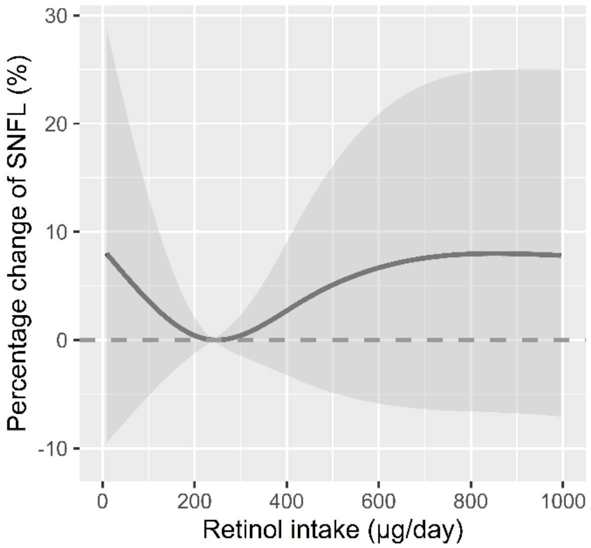

Figure S6. Relationship between dietary retinol and sNFL levels in participants with BMI>30 kg/m^2. Restriction cubic spline curves were used at the 5th, 35th, 65th and 95th percentiles of dietary retinol intake. The shaded part represents the 95% CI interval. Models were adjusted for age, gender, PIR, education, ethnicity, smoking, alcohol consumption, diabetes, hypertension, eGFR, MET, and dietary energy intake.

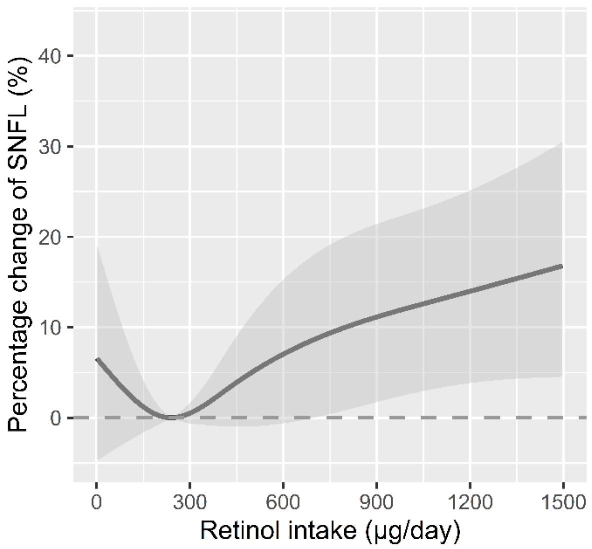

Figure S7. Association between dietary retinol and sNFL levels in participants without diabetes. Restriction cubic spline curves were used at the 5th, 35th, 65th and 95th percentiles of dietary retinol intake. The shaded part represents the 95% CI interval. Models were adjusted for age, gender, BMI, PIR, education, ethnicity, smoking, alcohol consumption, hypertension, eGFR, MET, and dietary energy intake.

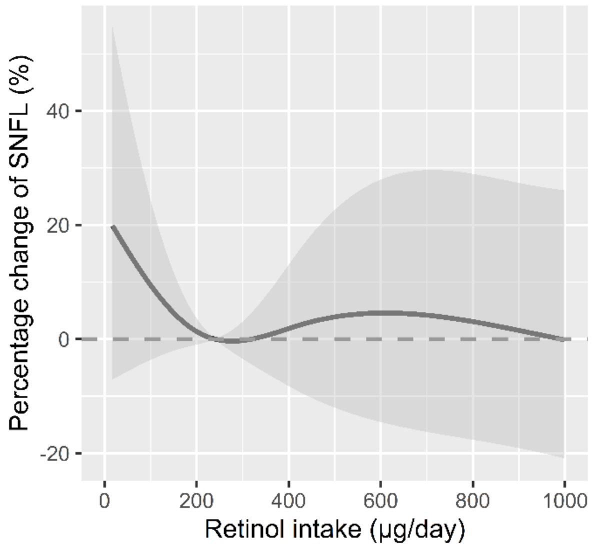

Figure S8. The relationship between dietary retinol and sNFL levels in diabetic patients. Restriction cubic spline curves were used at the 5th, 35th, 65th and 95th percentiles of dietary retinol intake. The shaded part represents the 95% CI interval. Models were adjusted for age, gender, BMI, PIR, education, ethnicity, smoking, alcohol consumption, hypertension, eGFR, MET, and dietary energy intake.

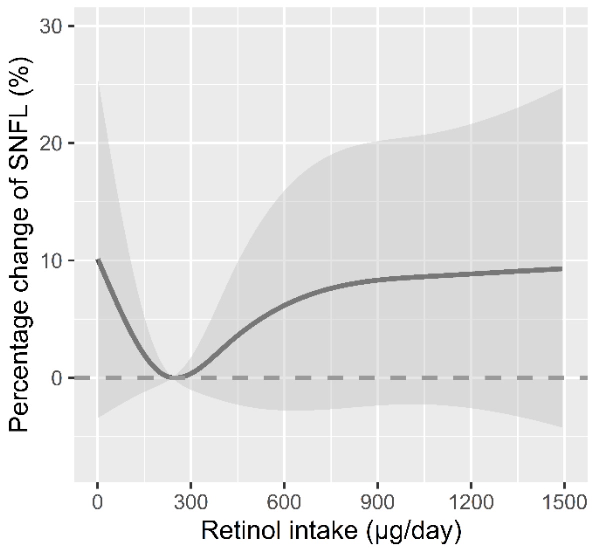

Figure S9. Association between dietary retinol and sNFL levels in participants with healthy renal function. Restriction cubic spline curves were used at the 5th, 35th, 65th and 95th percentiles of dietary retinol intake. The shaded part represents the 95% CI interval. Models were adjusted for age, gender, BMI, PIR, education, ethnicity, smoking, alcohol consumption, diabetes, hypertension, MET, and dietary energy intake.

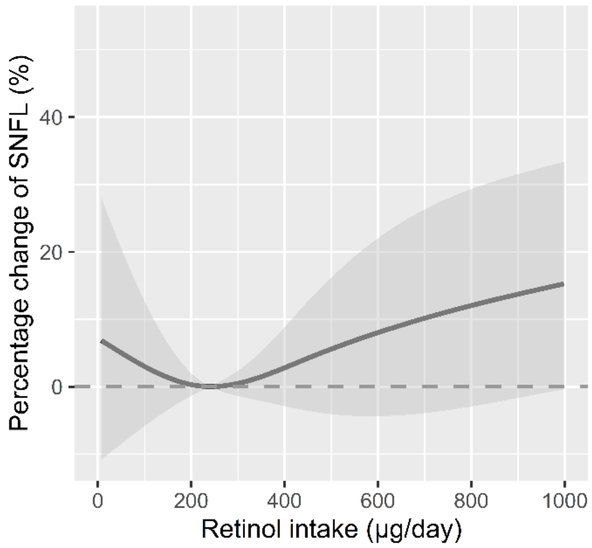

Figure S10. Association between dietary retinol and sNFL levels in participants with impaired renal function. Restriction cubic spline curves were used at the 5th, 35th, 65th and 95th percentiles of dietary retinol intake. The shaded part represents the 95% CI interval. Models were adjusted for age, gender, BMI, PIR, education, ethnicity, smoking, alcohol consumption, diabetes, hypertension, MET, and dietary energy intake.
